# Correlation between insulin-induced estrogen receptor methylation and atherosclerosis

**DOI:** 10.1186/s12933-016-0471-9

**Published:** 2016-11-10

**Authors:** Jia Min, Zhong Weitian, Cai Peng, Peng Yan, Zhang Bo, Wang Yan, Bai Yun, Wang Xukai

**Affiliations:** 1 Department of Cardiology, Daping Hospital, Third Military Medical University, Chongqing, 400042 China; 2 Department of Medical Genetics, College of Basic Medicine, Third Military, Medical University, Chongqing, 400038 China

**Keywords:** VSMC, Insulin, Estrogen receptor (ER), DNA methylation, Epigenetics

## Abstract

**Background:**

Hyperinsulinemia and insulin resistance have been recently recognized as an important cause of atherosclerosis. Clinical studies have also found that expression of the estrogen receptor is closely related to the incidence of atherosclerosis. This study investigate the effects of insulin and estrogen receptor α (ER-α) in atherosclerosis.

**Methods:**

Double knockout ApoE/Lepr mice were given intraperitoneal injections of insulin, and their aortae were harvested for hematoxylin-eosin staining and immunohistochemical analysis. In addition, vascular smooth muscle cells (VSMCs) were treated with insulin or infected with a lentivirus encoding exogenous ER-α, and changes in gene expression were detected by real-time polymerase chain reaction and western blotting. The methylation levels of the ER-α gene were tested using bisulfite sequencing PCR, and flow cytometry and EdU assay were used to measure VSMCs proliferation.

**Results:**

Our results showed that insulin can induce the formation of atherosclerosis. Gene expression analysis revealed that insulin promotes the expression of DNA methyltransferases and inhibits ER-α expression, while 5-aza-2′-deoxycytidine can inhibit this effect of insulin. Bisulfite sequencing PCR analysis showed that methylation of the ER-α second exon region increased in VSMCs treated with insulin. The results also showed that ER-α can inhibit VSMCs proliferation.

**Conclusions:**

Our data suggest that insulin promotes the expression of DNA methyltransferases, induces methylation of ER-α second exon region and decreases the expression of ER-α, thereby interfering with estrogen regulation of VSMCs proliferation, resulting in atherosclerosis.

**Electronic supplementary material:**

The online version of this article (doi:10.1186/s12933-016-0471-9) contains supplementary material, which is available to authorized users.

## Background

Atherosclerosis is a major cardiovascular disease affecting human health that is associated with a variety of factors such as obesity, abnormal lipid metabolism, hypertension, hyperinsulinemia and insulin resistance [1]. Insulin resistance leads to increased blood insulin levels, termed hyperinsulinemia, which has been confirmed by several clinical studies to be a predictor of cardiovascular disease [2, 3]. Abundant evidence also indicates that high concentrations of insulin can stimulate vascular smooth muscle cells (VSMCs) proliferation and migration [4, 5], which are currently thought to be the direct causes of atherosclerosis [6, 7].

An epidemiological study found that the incidence of cardiovascular diseases and insulin resistance are low in premenopausal women, with no significant differences between postmenopausal women and men, suggesting that endogenous estrogen in premenopausal women tends to reduce the risk of these diseases [8, 9]. Population-based observational studies have found that the application of estrogen in the early stage of postmenopausal women was associated with a reduced incidence of cardiovascular diseases and an improvement of insulin resistance. Estrogens exert their atheroprotective effects by binding to the estrogen receptor (ER), which regulates the transcription of many genes [10]. ER-α, ER-β and G protein-coupled estrogen receptor 1 (GPER1) are the three known receptors that mediate the effects of estrogen. The protective effect on vessels is retained in ER-β knockout mice but lost in ER-α knockout mice, suggesting that ER-α is essential for the protective effect of estrogen on the vasculature [11–13]. Lundholm et al. [14] found that ER-α activation with propyl pyrazole triol (PPT) reverses high fat diet-induced insulin resistance. Other researcher found that some male patients with ER-α deficiency exhibited insulin resistance, impaired glucose metabolism and hyperinsulinemia [15]. Estrogen can combine with ER-α to generate nitrogen monoxide (NO) in endothelial cells (ECs), which has a protective function on the vessels [1, 12, 16, 17] and suppresses smooth muscle cell proliferation by inhibiting the expression of extracellular signal-regulated kinase, c-Jun N-terminal kinase, P38, and other genes [18]. The expression of ER-α is decreased in the late stage of postmenopausal women, meaning that taking an estrogen supplement cannot reverse the risk of atherosclerosis, which is consistent with the findings of other studies [19–21]. Together, these data suggest that ER-α expression is one of the most important factors protecting premenopausal women from atherosclerosis.

Estrogen signaling is tightly intertwined with epigenetic regulation [22, 23], especially DNA methylation associated with gene repression. Methylation of ER genes is an important mechanism of reducing the expression of ER, and ER-α promoter methylation is increased in atherosclerotic patients and/or postmenopausal women [24]. At the same time, a growing body of evidence indicates the involvement of epigenetic alterations in atherosclerotic plaque formation and progression. The mechanism through which insulin promotes atherosclerosis also involves epigenetic modifications; high insulin levels reduce methylation of the leptin and adiponectin promoters, which contributes to atherosclerosis [25]. However, whether insulin induces ER-α methylation has not been investigated. To date, the nature of the relationship between hyperinsulinemia, ER-α expression and atherosclerosis is unclear.

In this study, we propose and test the hypothesis that high insulin levels can induce ER-α methylation and reduce ER-α expression, ultimately resulting in atherosclerosis. Double knockout ApoE/Lepr mice were given intraperitoneal injections of insulin for 12 weeks, and their aortae were harvested for hematoxylin–eosin (HE) staining and immunohistochemical analysis to investigate the formation of atherosclerotic plaques and differences in ER-α expression. We also tested ER-α and DNA methyltransferases (DNMTs) expression both in mRNA and protein levels in VSMCs treated with insulin. We also tested the methylation levels of the ER-α exon regions in VSMCs with or without insulin treatment. In addition, we measured the VSMCs proliferation after infection with a lentivirus encoding exogenous ER-α.

## Methods

### Materials

Specific pathogen free-grade ApoE/Lepr double knockout mice were purchased from The Jackson Laboratory (Bar Harbor, ME, USA). The lentivirus LV5-Esr1 (Rat) NM_012689.1 carrying an ER-α expression vector and the zero-load LV5NC (Suzhou Genepharma Co.,Ltd, Suzhou, China), a rat VSMC cell line, 5-Aza (Sigma-Aldrich, St. Louis, MO, USA), insulin for injection and insulin for cell culture (Roche, Basel, Switzerland), RIPA lysis buffer (Beyotime, Jiangsu, China), TriPure Isolation Reagent (Roche), anti-GAPDH antibody (PTSolution Biotechnology Co., Ltd, Guangzhou, China), anti-ER-α antibody (Merck Millipore, Bedford, MA, USA), anti-DNMT1 antibody (Santa Cruz Biotechnology, Dallas, TX, USA), anti-DNMT3a antibody (Abnova, Taibei, Taiwan), iTaq™ Universal SYBR^®^ Green Supermix (Bio-Rad, Hercules, CA, USA), and reverse transcription PrimeScript™ RT reagent kit with gDNA Eraser (Perfect Real Time, TaKaRa, Dalian, Japan) were used in this study.

### Animals and treatment

All animal work was approved by the Animal Care and Use Committee of the Third Military Medical University (Chongqing, China), and the methods were carried out in accordance with the approved guidelines. Mice were housed in a room with controlled temperature and 12 h light–dark cycle and had free access to water and standard chow. Eight-week-old ApoE/Lepr double knockout female mice (n = 8) were randomly divided in two groups: control group (N; n = 4) and insulin group (INS; n = 4), which received intraperitoneal injections of saline or insulin (4 IU) every other day for 12 weeks, respectively. Body weights and blood glucose were recorded once a week. At the end of the protocol, mice were euthanized by anesthesia for tissue harvesting.

### Cell culture and treatment

Rat VSMCs were cultured in Dulbecco’s modified Eagle’s medium (HyClone, Logan City, UT, USA) with 10% fetal bovine serum. Cells were treated with various combination of insulin (100 nM) and/or 5-Aza (10 μM), or infected with lentivirus carrying an ER-α expression vector and then were harvested for the indicated assays.

### Immunohistochemical staining

Fresh tissues were collected and fixed in 4% paraformaldehyde and embedded in paraffin. Tissue slides were obtained through serial sectioning and processing with standard procedures. The slides were stained with hematoxylin-eosin or ER-α antibody diluted at 1:100. After being washed, the slides were incubated with a biotinylated secondary antibody, and the color reaction was performed using ABC reagent (Zhongshan Co., Beijing, China) then counterstained with hematoxylin.

### Real-time PCR

Following the instructions of the PrimeScript™ RT reagent kit, 1 μg total RNA was reverse transcribed to cDNA. Quantitative PCR amplification was performed with a 10 µl final reaction mixture consisting of 0.5 µl of reverse transcription reaction, 0.1 µM of both the sense and antisense primers (Table 1) and the SYBR Green Master mix. The PCR conditions were an initial denaturation at 95 °C for 5 min followed by 40 cycles of PCR reaction: denaturation at 95 °C for 10 s; annealing at 58 °C for 20 s and elongation at 72 °C for 30 s. Gene expression was normalized to the corresponding Glyceraldehyde-3-phosphate dehydrogenase (*GAPDH*) levels and presented as the fold difference relative to *GAPDH*.

**Table 1:** Primer sequences

Primer	Sequence 5′ → 3′	
	Forward	Reverse
GAPDH	TACCCACGGCAAGTTCAA	GCCAGTAGACTCCACGACAT
DNMT1	ACCCCAGATGCTGACCAATG	ACTGATTGATTGGCCCCAGG
DNMT3a	AAGGAAGTTTACACCGACAT	TCTTACAGTTCTGGCACATT
ER-a	AGTGAAGCCTCAATGATGGG	GAGCAAGTTAGGAGCAAACAG
ER-a BSP*	GAGGTGTACGTGGATAATAGTAAGTT	CCAAATAATAAAACACCTAATAACCATAC

* Primers used for bisulfate sequencing PCR

### Western blotting

Cell pellets were harvested at the indicated times and were homogenized in ice-cold RIPA buffer (Beyotime) containing complete protease inhibitor cocktail. Total protein was resolved by sodium dodecyl sulfate polyacrylamide gel electrophoresis and transferred onto a nitrocellulose membrane. After blocking in 3% nonfat milk for 1 h, membranes were incubated with antibodies against total ER-α, DNMT1, DNMT3a, and *GAPDH*, followed by incubation with the corresponding secondary antibodies (Zhongshan Co.). The bands were visualized using the enhanced chemiluminescence system. The expression of individual proteins were normalized to *GAPDH*.

### Bisulfite sequencing PCR

The CpG island of ER-α situates in its second exon, the sequence was obtained by searching the University of California Santa Cruz (UCSC) Genome Bioinformatics website (http://genome.ucsc.edu/) and localizes in chr1:42669631–42670064. The BSP primers were designed by Methyl Primer Express v1.0 (Table 1). DNA was extracted from the rat VSMCs using Apoptosis, DNA Ladder Extracion Kit with Spin Column (Beyotime, Shanghai, China). Bisulfite modification of genomic DNA was performed by EZ DNA Methylation-Gold™ Kit (Zymo Research Corp, Orange, CA) (DNA sample was then treated with sodium bisulfate, converting unmethylated cytosine (C) to uracil and keeping methylated C intact). Then the bisulfate-modified DNA were amplified by PCR. The reaction conditions were as follows: pre-degeneration at 98 °C for 4 min, 94 °C for 45 s, 66 °C for 45 s, where the annealing temperature was reduced by 0.5 °C in each cycle and 72 °C for 1 min for a total of 20 cycles; 94 °C for 45 s, 56 °C for 45 s, and 72 °C for 1 min for a total of 20 cycles, with a final elongation step of 72 °C for 8 min. The PCR products were separated by electrophoresis in a 2% agarose gel and recycled by the gel extraction kit (Sigma-Aldrich), followed by linking with T-vectors (Suzhou Genepharma Co., Ltd, Suzhou, China) and white-blue plaque selection, 6–10 of positive clones were sequenced in each sample at BGI. The sequencing results were then imported into BIQ Analyzer software (a software tool for easy visualization and quality control of DNA methylation data from bisulfate sequencing, http://biq-analyzer.bioinf.mpi-inf.mpg.de/) for comparing and calculating the methylation ratio.

### Lentiviral infection

Approximately 1 × 10^4^ VSMCs were inoculated in culture flasks with 50 µl of LV5-Esr1 or LV5NC to make up the final volume of 2 ml complete medium. The medium was changed after 24 h, and fluorescence was observed after 72 h.

### EdU assay and flow cytometry

VSMCs were inoculated in 96-well plates, approximately 5000/well. Then following the manufacturer’s instructions of the EdU (5-ethynyl-29-deoxyuridine) assay kit (Ribobio, Guangzhou, China), cells proliferation was calculated. The cells were exposed to 50 µM of EdU for 2 h at 37 °C. The cells were then fixed with 4% formaldehyde for 30 min at room temperature and treated with 0.5% Triton X-100 for 10 min at room temperature for permeabilization. After wash with PBS, the cells were treated with 100 µl of 1 × Apollo^R^ reaction cocktail for 30 min. Subsequently, the DNA contents of each well of cells were stained with 100 µl of 1 × Hoechst 33,342 for 10 min and visualized under a fluorescent microscope (Olympus, Japan).

VSMCs were collected, resuspended in 70% ethanol, and stored at −20 °C overnight. Prior to mounting, the cells were thrice rinsed with phosphate buffer saline (PBS), stained with propidium iodide, and incubated at room temperature in the dark, then analyzed using flow cytometry.

### Statistical analysis

The data are presented as mean ± SD. For the comparison between multiple groups, quantitative data were analyzed using one-way ANOVA and Spearman rank correlation analysis, and qualitative data were analyzed using Chi square test. Values less than P < 0.05 were considered significant.

## Results

### Insulin decreased ER-α expression in VSMCs in the atherosclerosis plaque of mice

**Fig. 1:**
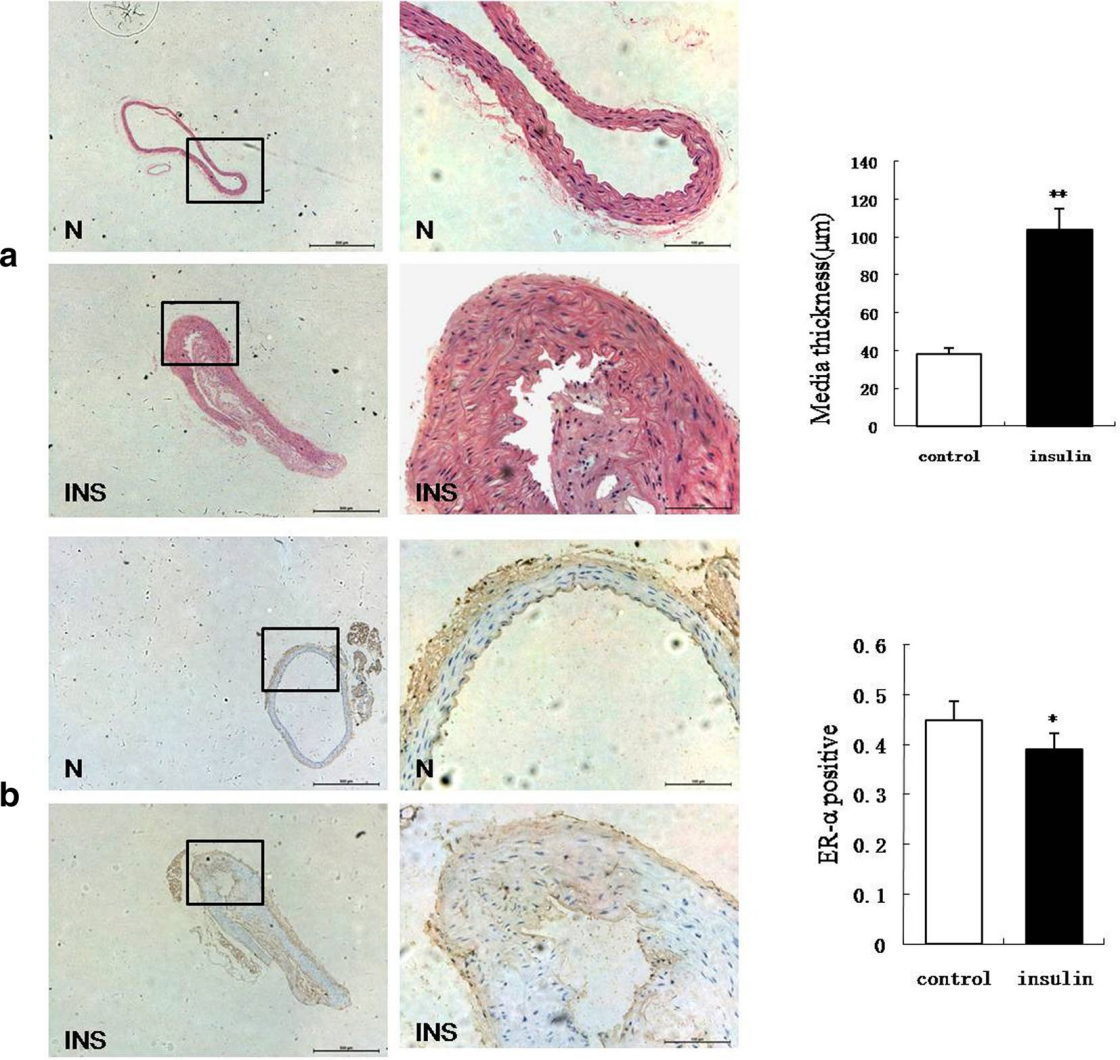
Representative hematoxylin-eosin or ER-α antibody stained histological sections of aorta from insulin treatment group and control group

Mice in the experimental group showed significant atherosclerotic plaques (mean plaque area: 104,733 ± 11.3 × 10^3^ µm^2^), thickened walls, and a disordered arrangement of nuclei after the 12-week treatment of intraperitoneal insulin injections compared with the control group (Fig. 1a; P < 0.01). Immunohistochemistry revealed that the expression of ER-α protein in the vascular smooth muscle nuclei was significantly decreased in the experimental group compared with the control group (Fig. 1b; P < 0.05). Moreover, the insulin level and HOMA-IR of the experimental group were higher significantly compare with the control group. But there were no differences in mean body weight or blood glucose between the two groups (Additional file 1: Table S1, Additional file 2: Figure S1). Therefore, we hypothesized that insulin induced atherosclerosis and was responsible for the decreased expression of ER-α.

### Insulin inhibited ER-α expression and promoted DNMTs expression in VSMCs

**Fig. 2:**
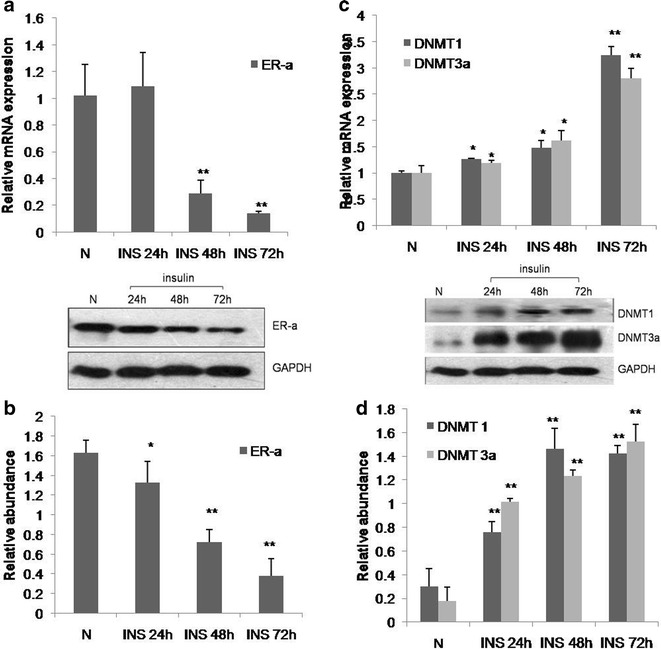
Effects of insulin inhibited ER-α expression and promoted DNMTs expression in VSMCs

To directly test whether insulin inhibited ER-α expression, we treated VSMCs with insulin for 24, 48 and 72 h. Quantitative reverse transcriptase polymerase chain reaction (RT-PCR) analysis confirmed that insulin treatment significantly decreased the mRNA level of ER-α compared with the control group (Fig. 2a; P < 0.01). To further confirm this effect of insulin, ER-α protein was measured by western blot. The results showed that insulin treatment reduced the expression of ER-α compared with the control group (Fig. 2b; P < 0.01). These data clearly indicate that insulin inhibits ER-α expression in VSMCs.

It has been reported that the majority of cellular DNMT activity is contributed by DNMT1, DNMT3a and DNMT3b. DNMT3a and DNMT3b have similar functions, so we tested the expression of DNMT1 and DNMT3a to determine whether their expression correlated with the inhibition of ER-α expression by insulin. The effect of insulin on DNA methyltransferase expression was tested using RT-PCR and western blot. The results showed that DNMT1 and DNMT3a were significantly increased both in mRNA (Fig. 2c) and protein (Fig. 2d) levels in the insulin treatment group compared with the control group (P < 0.01), further demonstrating that insulin epigenetically inhibited ER-α expression.

### 5-aza-2′-deoxycytidine (5-Aza) inhibited the insulin-dependent down-regulation of ER-α

**Fig. 3:**
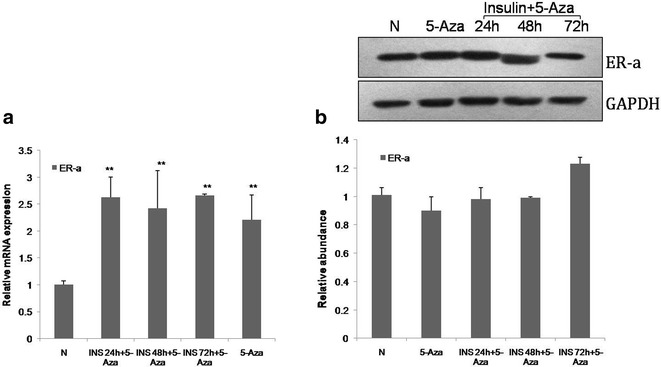
Effects of 5-Aza inhibited the insulin-dependent down-regulation of ER-α

To determine if the regulation of ER-α expression by insulin involved epigenetics we treated VSMCs with 5-Aza and insulin and tested the expression of ER-a by RT-PCR and western-blot. The mRNA level of ER-α was increased when VSMCs were treated with 5-Aza alone or in combination with insulin compared with the control group (Fig. 3a; P < 0.01). And the protein level of ER-α was not decreased when VSMCs were treated with 5-Aza and insulin compare with control (Fig. 3b; P > 0.05). The data suggest that 5-Aza can inhibit the insulin-induced suppression of ER-α.

### Effect of insulin on the methylation status of the ER-α second exon region

**Fig. 4:**
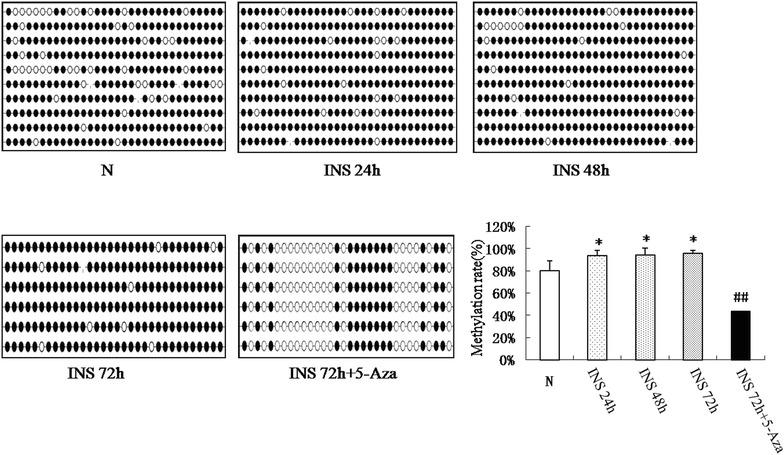
Methylation status of the ER-α second exon by bisulfite sequencing PCR in the insulin treatment group and the control group

DNMTs inhibits gene expression by promoting DNA methylation, and the results of bisulfite sequencing PCR showed that the extent of methylation in the ER-α second exon region was significantly increased in the insulin treatment group compared with the control group (Fig. 4; P < 0.05). Moreover, the extent of methylation in the ER-α second exon region was significantly decreased after VSMCs were treated with 5-Aza compared with the insulin group (P < 0.01). These data suggest that insulin signaling promotes the expression of DNMTs, which promotes methylation of the ER-α second exon, and ultimately inhibits ER-α expression.

### ER-α inhibited VSMCs proliferation

**Fig. 5:**
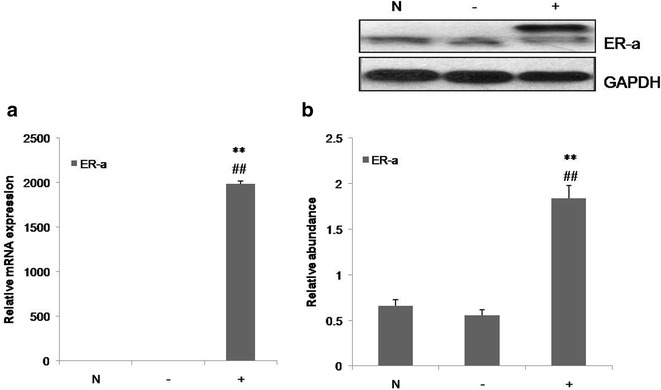
ER-α expression in VSMCs infected with LV5-Esrl (the lentivirus carrying an ER-α expression vector) and in the control and zero-load lentiviral infection groups

**Fig. 6:**
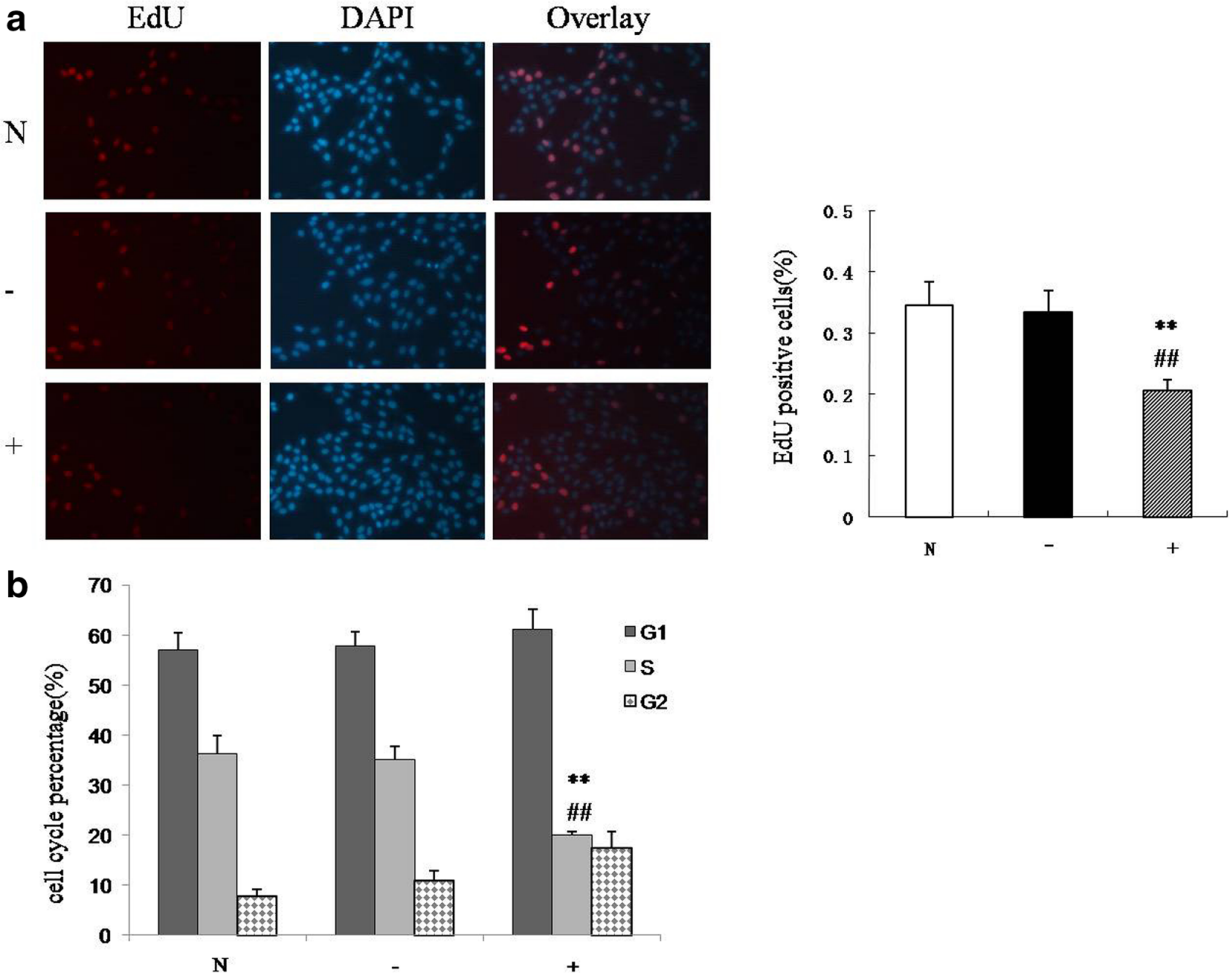
The effect of ER-α in VSMCs proliferation

To study the effect of ER-α in VSMC proliferation we over-expressed ER-α by transducing a lentiviral expression vector containing ER-α. ER-α expression in VSMCs was then tested by RT-PCR and western blot, and the results showed that ER-α expression was significantly higher in VSMCs infected with LV5-Esrl, a lentivirus carrying a V5-tagged ER-α expression vector, than in the control and zero-load lentiviral infection groups (Fig. 5; P < 0.01). The ER-α mRNA and protein levels did not differ significantly in VSMCs in the control and zero-load viral infection groups. The results of EdU assay showed that ER-α inhibited VSMCs proliferation (Fig. 6a; P < 0.01), and cell cycle analysis by flow cytometry revealed that the proportion of S-phase cells was significantly decreased in the LV5-Esrl-infected group compared with the control and zero-load virus-infected groups, and the differences were statistically significant (Fig. 6b; P < 0.01). These results suggest that ER-α inhibits VSMCs proliferation.

## Discussion

In this study, we found significant atherosclerotic plaques and decreased ER-α expression in aortae of double knockout ApoE/Lepr mice treated with insulin, and we demonstrated that insulin signaling leads to epigenetic modifications that promote atherosclerosis, including inducing hypermethylation of the ER-α second exon region, which subsequently decreased ER-α mRNA and protein expression. We also demonstrated that VSMCs proliferation is inhibited by exogenous ER-α expression.

Hyperinsulinemia is now considered a risk factor of cardiovascular diseases such as hypertension, atherosclerosis, and intimal injury. Although there are abundant studies about the mechanism of insulin signaling pathway to atherosclerosis [26], the specific pathogenic mechanism is still unclear, it is generally believed to be associated with hyperinsulinemia-induced phenotypic modulation of VSMCs. Lineage-tracing studies have illustrated that significant plasticity is maintained in the VSMCs of adult animals [25, 26], which might perform reversible phenotypic modulation when subjected to vascular injury, cytokines, growth factors or diseases. These shifts in phenotype tend to be from contractile to synthetic types, resulting in enhanced VSMCs proliferation and migration, which can lead to atherosclerosis [26–29]. Previously, our study had found that insulin promotes VSMCs proliferation and migration via microRNA-208-mediated downregulation of p21 and inducing dopamine D_1_ receptor dysfunction, and activation of the dopamine D_4_ receptor suppresses the effect of insulin on VSMCs [30–32]. Our present in vivo experiments confirmed that insulin can promote atherosclerotic plaque formation and VSMCs proliferation. The results of HE staining showed thickened aorta walls, disordered nuclei arrangements and significant atherosclerotic plaques that are consistent with previous studies.

Human ER-α is considered an atheroprotective gene that regulates the beneficial effects of estrogen on endothelial cells and VSMCs. ER-α expression was found to be significantly decreased in aortic VSMCs from patients with atherosclerosis compared with normal control subjects, and accompanied by increased methylation of the ER-α promoter [33, 34]. However, the mechanisms underlying these changes in ER-α promoter methylation are unknown, but may be due to the impact of the environment and diet according to the epigenetic regulation theory.

Insulin is an atherogenic molecule, and meanwhile, is part of the complex external environment that influences cell behaviors and phenotypes, and a change in insulin concentration has been confirmed to alter DNA methylation patterns. It can lead to different methylation patterns of the same genes in different cell types as well as cause different methylation patterns of various genes within the same cell type [35–37]. However, insulin-induced methylation of ER-α in VSMCs had not been previously reported, so we tested ER-α expression in the aorta wall of the double knockout ApoE/Lepr mice primarily. The results showed that ER-α expression was lower in the insulin-treated group than the control group, suggesting that insulin-induced atherosclerosis was mediated by decreased ER-α expression. We subsequently validated these results in vitro using a rat VSMC line. We found that ER-α expression was also decreased in the VSMCs treated with insulin.

ER-α expression is related to DNA methylation (cytosine methylation in CpG dinucleotides), which is typically associated with reduced gene transcription through a chemically stable epigenetic modification carried out by DNMTs [38]. Hence, we tested DNMT1 and DNMT3a expression in VSMCs treated with insulin. The results showed that DNMT1 and DNMT3a mRNA and protein expression were both increased after insulin treatment. We also found that the effect on ER-α expression could be inhibited by the DNMT inhibitor, 5-Aza; ER-α expression was increased in VSMCs treated with insulin and 5-Aza compared to cells treated with insulin alone. These data further indicate that the insulin-induced ER-α repression was through DNA methylation. We also measured the extent of ER-α second exon region methylation using bisulfite sequencing PCR. These results confirmed previous observations and directly showed that DNA methylation levels in the ER-α second exon region were increased in the insulin treatment group, but were significantly decreased after co-treatment with 5-Aza. These results also highlighted that the baseline methylation level of ER-α in the control group was high. The VSMCs we used were senescing, and perhaps this could explain the high baseline level of ER-α methylation, which was consistent with the previous study concluded that methylation of the ER gene is associated with aging [24]. To determine whether ER-α inhibits VSMCs proliferation, we tested VSMCs proliferation after over-expressing ER-α. We found that VSMCs proliferation was lower after infection with LV5-Esrl.

Atherosclerosis is a chronic inflammatory condition of the vessel wall, and a large number of studies have investigated the molecular mechanisms underlying plaque formation and progression [39]. Recently, a growing body of evidence has indicated the involvement of epigenetic alterations in plaque formation and progression and in other atherosclerosis-related diseases. To date, epigenetic modifications have been reported to regulate some of the genes implicated in extracellular matrix formation, inflammation, and cell proliferation, which are involved in atherosclerosis [40–42]. For example, the NOS3 gene (encoding the endothelial eNOS) promoter in endothelial cells is hypomethylated, but hypermethylation in smooth muscle cells [43, 44]. The main epigenetic mechanisms include DNA methylation, histone modifications, and RNA-based mechanisms regulating gene silencing and activation. This study revealed, for the first time, that insulin promotes atherosclerosis through epigenetic modifications, specifically, by inducing hypermethylation of the ER-α second exon region, which reduces ER-α expression and promotes atherosclerosis. Our data reveal a new mechanism and provide new evidence for insulin promoting atherosclerosis. This work will help us find new therapeutic targets for atherosclerosis and choose better therapeutic regimens for patients with diabetes and menopause problems.

## Conclusion

High blood insulin concentration is an important factor in the incidence of atherosclerosis, especially for women in the latter stages of menopause. Our manuscript is the first report that high levels of insulin lower ER-α expression via inducing hypermethylation of the ER-α second exon region. This decreases estrogen signaling and allows for upregulated VSMCs proliferation. Therefore, in addition to controlling blood sugar levels, controlling insulin levels is essential for patients with type 2 diabetes mellitus, because of this link with atherosclerosis-associated diseases. Nevertheless, there are some unresolved questions for us to elucidate. First, we are not sure if there will be the same significant change in ER-α hypermethylation in VSMCs of atherosclerotic patients treated with insulin; second, the effect of insulin on ER-α transcriptional activity in VSMCs has not been studied. All these questions and more will be the focus of our further studies.

## Additional files

**Additional file 1: Table S1.** The mean body weight, blood glucose, insulin level and HOMA-IR of the ApoE/Lepr double knockout mice after 12 weeks.

**Additional file 2: Figure S1.** The mean body weight and blood glucose of the ApoE/Lepr double knockout mice every week.

## Abbreviations

ER-α: estrogen receptor α
VSMCs: vascular smooth muscle cells
PCR: polymerase chain reaction
GPER1: G protein-coupled estrogen receptor 1
NO: nitrogen monoxide
ECs: endothelial cells
HE: hematoxylin–eosin
DNMT: DNA methyltransferase
PBS: phosphate buffer saline
