# Supplementary material for: Correlation between insulin-induced estrogen receptor methylation and atherosclerosis
Source: Cardiovasc Diabetol. 2016 Nov 10;15:156. doi: 10.1186/s12933-016-0471-9 (PMC5105242; doi:10.1186/s12933-016-0471-9)
Supplement: Supplementary file 1 — Additional file 1: Table S1. The mean body weight, blood glucose, insulin level and HOMA-IR of the ApoE/Lepr double knockout mice after 12 weeks. [file 12933_2016_471_MOESM1_ESM.doc]

Table S1. The mean body weight, blood glucose, insulin level and HOMA-IR of the ApoE/Lepr double knockout mice after 12 weeks.

|  | **Control group**  **Mean±SD** | **Insulin group**  **Mean±SD** |
| --- | --- | --- |
| Body weight (g) | 53.75 ±5.38 | 53 ±4.32 |
| Blood glucose (mM) | 7.59 ±0.4 | 7.5 ±0.55 |
| Insulin level (mIU/L) | 3.4 ±0.35 | 5.94 ±0.51 * |
| HOMA-IR | 1.14 ±0.09 | 2.07 ±0.22* |
