# Supplementary figures and images for: Correlation between insulin-induced estrogen receptor methylation and atherosclerosis
Source: Cardiovasc Diabetol. 2016 Nov 10;15:156. doi: 10.1186/s12933-016-0471-9 (PMC5105242; doi:10.1186/s12933-016-0471-9)

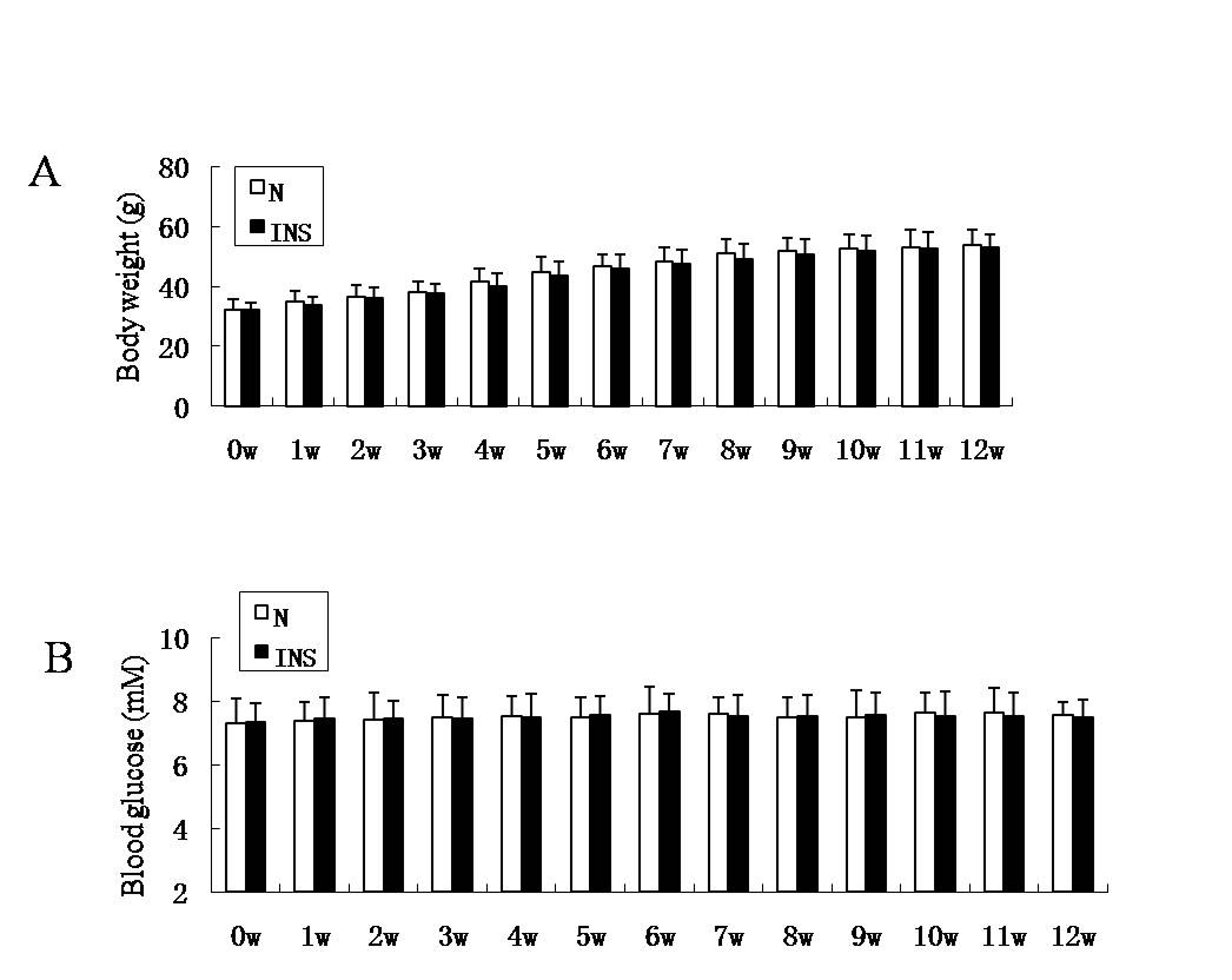

Supplement: Supplementary file 2 — Additional file 2: Figure S1. The mean body weight and blood glucose of the ApoE/Lepr double knockout mice every week. [file 12933_2016_471_MOESM2_ESM.jpg]
